# Collagenous Colitis Associated with Protein Losing Enteropathy in a Toddler

**DOI:** 10.1155/2014/209624

**Published:** 2014-08-20

**Authors:** Osama F. Almadhoun, Philip J. Katzman, Thomas Rossi

**Affiliations:** ^1^University of Kansas Medical Center, KU Pediatrics, Mail Stop 4004, 3901 Rainbow Boulevard, Kansas City, KS 66160, USA; ^2^University of Rochester Medical Center, Golisano Children Hospital, 601 Elmwood Avenue, Rochester, NY 14642, USA

## Abstract

Collagenous mucosal inflammatory disease is a rare gastrointestinal disorder that involves the columnar lining of gastric and intestinal mucosa and is characterized by a distinct subepithelial collagen deposition. Recent clinical and pathological evidence have indicated that collagenous mucosal inflammatory disease can be extensive disease that may concomitantly involve several gastrointestinal sites at the same time. This entity, however, occurs infrequently in children. It is even less common to find concomitant depositions of collagen in the mucosa of gastrointestinal sites other than the colon. Only two cases in pediatric literature reported concomitant involvement, one with gastric and colonic involvement and the other one with gastroduodenocolitis. We are reporting a 15-month-old boy who presented with severe diarrhea and diffuse edema secondary to hypoalbuminemia. Further testing documented protein losing enteropathy (PLE) associated with collagenous colitis.

## 1. Case Report

**Figure 1:**
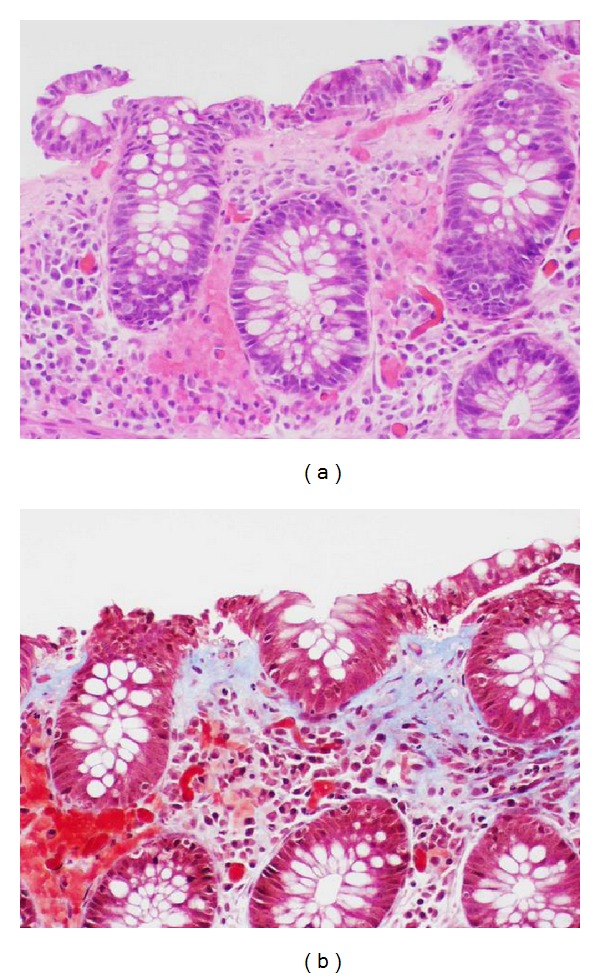
Colonic mucosa with thickened subsurface collagen deposition ((a) hematoxylin-eosin; (b) Masson trichrome, original magnification ×200).

**Figure 2:**
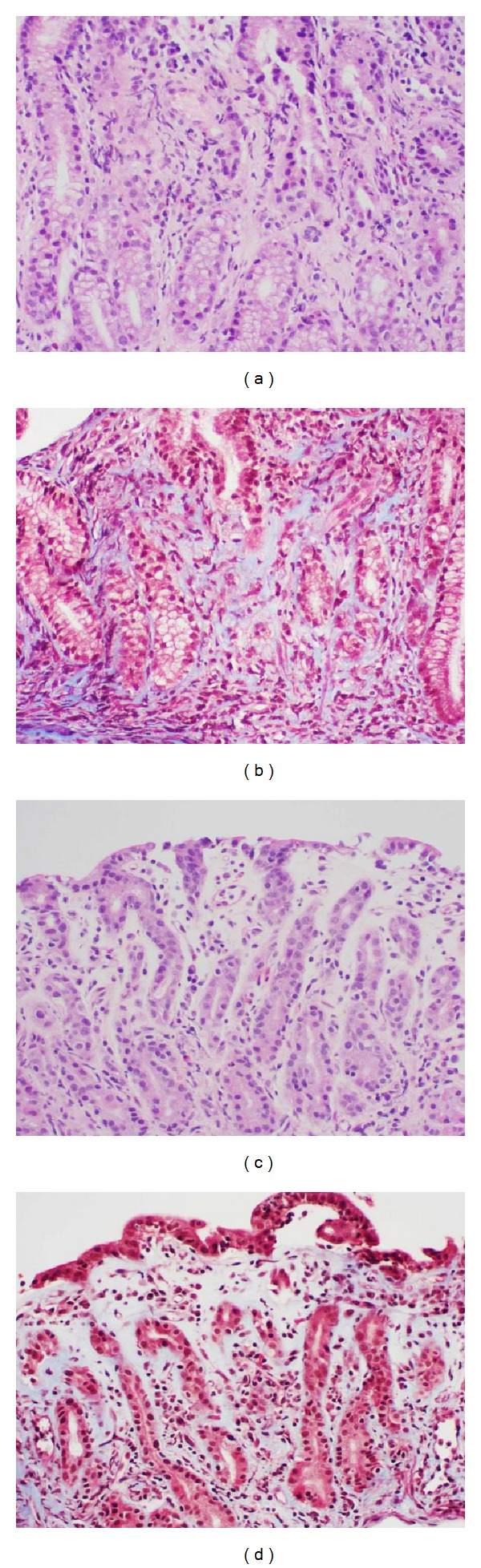
Gastric mucosa with dispersed collagen in lamina propria but not with distinct subsurface collagen deposition ((a), (b) antrum; (c), (d) body; (a), (c) hematoxylin-eosin, (b), (d) Masson trichrome, original magnification ×200).

**Figure 3:**
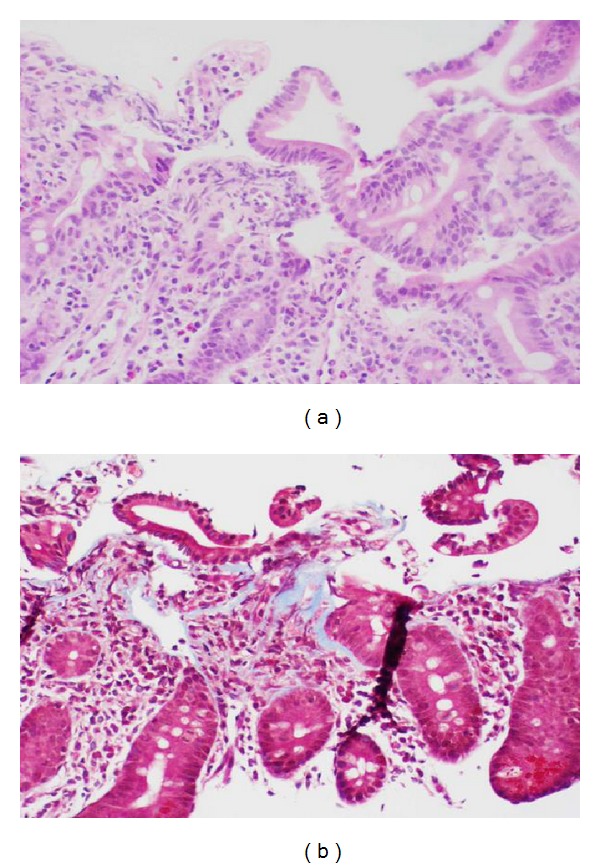
Duodenal mucosa with only scant foci of subsurface collagen deposition not diagnostic of collagenous duodenitis ((a) hematoxylin-eosin; (b) Masson trichrome, original magnification ×200).

A 15-month-old male, with a past medical history significant for microcornea, was admitted to our hospital with progressive peripheral edema for two weeks. Parents reported 4-5 explosive diarrheal bowel movements per day for approximately 4 weeks prior to presentation. The bowel movements were described as yellow-green in color, with associated “little globules,” liquid to pasty in consistency without blood or mucus. Shortly before admission, he began vomiting after almost every meal. On physical exam he was ill-appearing, afebrile, with significant facial and upper and lower limb edema. Laboratory investigations revealed the following: white blood cells, 14.5/mm^3^; C-reactive protein, <0.03 mg/dL; total protein, 3.6 g/dL; plasma albumin, 2.4 mg/dL; no abnormalities in liver, renal, and thyroid function tests; normal results of urinalysis and no proteinuria. Immunoglobulin A tissue transglutaminase, Epstein-Barr virus profile, and Cytomegalovirus profile were all negative. Stool for bacterial and viral cultures, Giardia antigen, white cells, and blood occult were also negative. Stool for alpha-1 antitrypsin was elevated at >1.33 mg/g (normal <0.62 mg/g), consistent with protein losing enteropathy. Upper endoscopy showed edematous antral and duodenal mucosa. The rectal and sigmoid mucosa appeared normal on flexible sigmoidoscopy. Biopsies from the colon and rectal mucosa showed patchy increased subepithelial bands of collagen of variable thicknesses consistent with collagenous colitis. The collagen deposition was highlighted with Masson trichrome staining (Figure 1). The gastric biopsy contained chronic gastritis with increased fibrosis and collagen deposition but no clear subepithelial collagen band (Figure 2). The duodenal biopsy showed foci of possible increased collagen in the mucosa, but not enough to be diagnostic for duodenal involvement of collagenous mucosal inflammatory disease (Figure 3). Budesonide was then initiated at 3 mg per day for 5 days but he continued with diarrhea and protein loss requiring parenteral nutrition replacement. Intravenous methylprednisolone was then added at 2 mg/kg/day and a marked improvement in his symptoms was noticed. He gradually tolerated oral intake and was weaned off total parenteral nutrition over a one-week period. He was discharged home on prednisolone 1 mg/kg/dose in addition to 3 mg per day of budesonide. He continued to do well after weaning off prednisone over two months. An attempt to discontinue budesonide led to worsening diarrhea and a requirement for reinduction for remission with oral steroid along with budesonide. He was then maintained on 3 mg of budesonide per day and continued to do well clinically. Upper endoscopy and colonoscopy were repeated 5 months later and showed persistent collagenous colitis without gastric or duodenal involvement. Biopsies at endoscopy and colonoscopy one year after presentation were normal on maintenance budesonide. He continued to do clinically well thereafter with no GI symptoms.

## 2. Discussion 

Collagenous inflammatory mucosal disease is a relatively uncommon disorder [1]. Collagenous colitis (CC) is the most commonly known presentation of this disease and was first described in 1976 [2] in adults and in 1989 in children [3–5]. Typically, it is a disease of middle-aged women with a clinical picture characterized by nonbloody chronic watery diarrhea and histology showing a thickened subepithelial collagenous band [1].

There is growing evidence in adult literature that collagenous inflammatory mucosal disease is a more extensive pathologic process that concomitantly might involve several other gastrointestinal sites and not only the colon [6, 7]. Searching the pediatric literature revealed more than twenty-four reported patients with collagenous gastritis [8], more than nine cases of collagenous colitis [3–5, 9–14], one with gastrocolonic involvement [11], and one with involvement of gastric, duodenal, and colonic mucosa [15]. Abdominal pain, weight loss, anemia, and fatigue were more likely to occur in patients with isolated collagenous gastritis, while diarrhea was the predominant symptom in the case of colonic involvement [8].

Excessive enteric protein loss is not a commonly recognized manifestation of collagenous inflammatory mucosal disease. There is only one reported case in the literature of a 15-month-old with collagenous gastroduodenocolitis who presented with profound diarrhea, anemia, and low albumin [15]. In adults, only 2 cases of CC associated with PLE have been reported [16, 17]. PLE was not part of the clinical presentations in those adult patients who had gastric or small intestinal involvement with CC [6, 7].

The exact mechanism of gastrointestinal protein loss in this condition is unknown, but abnormalities in the surface epithelium, superficial capillaries, and pericryptal fibroblast have been hypothesized [17]. Corticosteroids, budesonide, and 5-ASA have been all successfully used in the treatment of collagenous inflammatory mucosal disease [8]. Resolution varies and relapse frequently occurs. Our case responded to budesonide and methylprednisolone initially but relapsed after discontinuation. Remission was then reestablished with the introduction of both prednisolone and budesonide, and then patient was maintained on low dose budesonide. Biopsies procured during budesonide treatment were normal as well as stool alpha-1 antitrypsin level.

Although the initial duodenal biopsy had features that were suggestive of but not diagnostic for enteral involvement of the collagenous mucosal inflammatory disease, additional sampling of the duodenum and terminal ileum may have been diagnostic. The presence of chronic gastritis with fibrosis in the lamina propria may also obscure some degree of collagenous gastritis that may have been present in our case.

In conclusion, we have described an unusual case of a 15-month-old boy with collagenous colitis who presented with PLE in the absence of any clear risk factors and who had complete and maintained clinical remission on anti-inflammatory medications. Patients with collagenous inflammatory mucosal disease may be at risk of PLE and this entity should be considered in the differential diagnosis while working up children with diarrhea and PLE.
